# Supplementary material for: Causal Relationship between Aging and Anorexia Nervosa: A White-Matter-Microstructure-Mediated Mendelian Randomization Analysis
Source: Biomedicines. 2024 Aug 16;12(8):1874. doi: 10.3390/biomedicines12081874 (PMC11351342; doi:10.3390/biomedicines12081874)
Supplement: Supplementary file 1 [file biomedicines-12-01874-s001.zip › Supplementary Figure/Figure S4/Singe SNP plot of MR analysis for TL to ubm-b-1953.pdf]

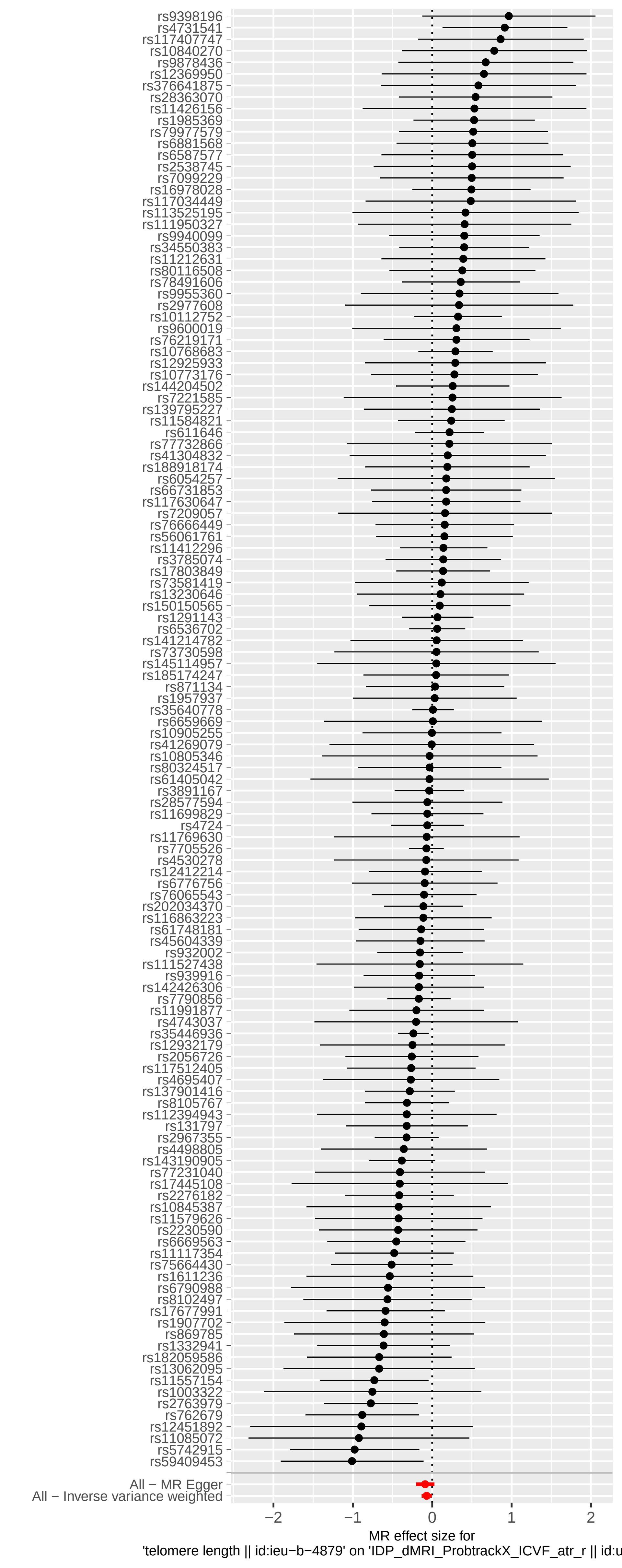
rs9398196
rs4731541
rs117407747
rs10840270
rs9878436
rs12369950
rs376641875
rs28363070
rs11426156
rs1985369
rs79977579
rs6881568
rs6587577
rs2538745
rs7099229
rs16978028
rs117034449
rs113525195
rs111950327
rs9940099
rs34550383
rs11212631
rs80116508
rs78491606
rs9955360
rs2977608
rs10112752
rs9600019
rs76219171
rs10768683
rs12925933
rs10773176
rs144204502
rs7221585
rs139795227
rs11584821
rs611646
rs77732866
rs41304832
rs188918174
rs6054257
rs66731853
rs117630647
rs7209057
rs76666449
rs56061761
rs11412296
rs3785074
rs17803849
rs73581419
rs13230646
rs150150565
rs1291143
rs6536702
rs141214782
rs73730598
rs145114957
rs185174247
rs871134
rs1957937
rs35640778
rs6659669
rs10905255
rs41269079
rs10805346
rs80324517
rs61405042
rs3891167
rs28577594
rs11699829
rs4724
rs11769630
rs7705526
rs4530278
rs12412214
rs6776756
rs76065543
rs202034370
rs116863223
rs61748181
rs45604339
rs932002
rs111527438
rs939916
rs142426306
rs7790856
rs11991877
rs4743037
rs35446936
rs12932179
rs2056726
rs117512405
rs4695407
rs137901416
rs8105767
rs112394943
rs131797
rs2967355
rs4498805
rs143190905
rs77231040
rs17445108
rs2276182
rs10845387
rs11579626
rs2230590
rs6669563
rs11117354
rs75664430
rs1611236
rs6790988
rs8102497
rs17677991
rs1907702
rs869785
rs1332941
rs182059586
rs13062095
rs11557154
rs1003322
rs2763979
rs762679
rs12451892
rs11085072
rs5742915
rs59409453
All − MR Egger
All − Inverse variance weighted
−2
−1
0
1
2
MR effect size for
'telomere length || id:ieu−b−4879' on 'IDP_dMRI_ProbtrackX_ICVF_atr_r || id:u
